# Long-Term Exposure to PM_2.5_, Facemask Mandates, Stay Home Orders and COVID-19 Incidence in the United States

**DOI:** 10.3390/ijerph18126274

**Published:** 2021-06-10

**Authors:** Fang Fang, Lina Mu, Yifang Zhu, Jianyu Rao, Jody Heymann, Zuo-Feng Zhang

**Affiliations:** 1 Department of Epidemiology, Fielding School of Public Health, University of California at Los Angeles (UCLA), Los Angeles, CA 90095, USA; clairyff@ucla.edu (F.F.); jrao@mednet.ucla.edu (J.R.); 2 Department of Epidemiology and Environmental Health, School of Public Health and Health Professions, University at Buffalo, The State University of New York, Buffalo, NY 14214, USA; linamu@buffalo.edu; 3 Department of Environmental Health Science, University of California at Los Angeles (UCLA), Los Angeles, CA 90095, USA; yifang@ucla.edu; 4 Institute of the Environment and Sustainability, University of California at Los Angeles (UCLA), Los Angeles, CA 90095, USA; 5 Department of Pathology and Laboratory Medicine, David Geffen School of Medicine, University of California at Los Angeles (UCLA), Los Angeles, CA 90095, USA; 6 WORLD Policy Analysis Center, University of California at Los Angeles (UCLA), Los Angeles, CA 90095, USA; jody.heymann@ph.ucla.edu; 7 Jonsson Comprehensive Cancer Center, University of California at Los Angeles (UCLA), Los Angeles, CA 90095, USA; 8 Center for Human Nutrition, Department of Medicine, David Geffen School of Medicine, University of California at Los Angeles (UCLA), Los Angeles, CA 90095, USA

**Keywords:** particulate matter, COVID-19, facemasks, stay-home orders, nation-wide study

## Abstract

Long-term PM_2.5_ exposure might predispose populations to SARS-CoV-2 infection and intervention policies might interrupt SARS-CoV-2 transmission and reduce the risk of COVID-19. We conducted an ecologic study across the United States, using county-level COVID-19 incidence up to 12 September 2020, to represent the first two surges in the U.S., annual average of PM_2.5_ between 2000 and 2016 and state-level facemask mandates and stay home orders. We fit negative binomial models to assess COVID-19 incidence in association with PM_2.5_ and policies. Stratified analyses by facemask policy and stay home policy were also performed. Each 1-µg/m^3^ increase in annual average concentration of PM_2.5_ exposure was associated with 7.56% (95% CI: 3.76%, 11.49%) increase in COVID-19 risk. Facemask mandates and stay home policies were inversely associated with COVID-19 with adjusted RRs of 0.8466 (95% CI: 0.7598, 0.9432) and 0.9193 (95% CI: 0.8021, 1.0537), respectively. The associations between PM_2.5_ and COVID-19 were consistent among counties with or without preventive policies. Our study added evidence that long-term PM_2.5_ exposure increased the risk of COVID-19 during each surge and cumulatively as of 12 September 2020, in the United States. Although both state-level implementation of facemask mandates and stay home orders were effective in preventing the spread of COVID-19, no clear effect modification was observed regarding long-term exposure to PM_2.5_ on the risk of COVID-19.

## 1. Introduction

A novel coronavirus disease (COVID-19) was first discovered in Wuhan, China in December 2019 [1], and on 11 March 2020, a global pandemic was declared by the World Health Organization (WHO) [2]. As of 12 September 2020, COVID-19 has infected 6,353,677 people in the United States [3]. To avoid the human-to-human transmission of the pathogen, the U.S. Centers for Disease Control and Prevention (CDC) recommends social distancing, face masking, and good hygiene practices [4]. Each state also implements different policies in order to slow down the spread of the disease [5]. A meta-analysis including 21 studies showed the efficacy of face masks in preventing respiratory virus transmission. The protective effect of facemask use against respiratory virus infection was 64% and a 47% risk reduction was observed among non-healthcare workers. Among the studies included, one study observed a 96% reduction of COVID-19 risk among Chinese healthcare workers using facemasks [6]. A recent study also demonstrated face coverings as effective preventive measures in slowing down the viral transmission via droplets by mimicking cough-generated airborne particles in an indoor environment. The study showed that surgical and K95/KN95 masks reduced cough droplets dramatically [7]. By utilizing COVID-19 cases from 190 countries between 23 January 2020, and 13 April 2020, non-pharmaceutical interventions, such as mandatory masks, quarantine, distancing and traffic restriction, were inversely associated with the reproduction number of COVID-19. The reductions in reproduction numbers were −15.14% (from −21.79% to −7.93%) for mandatory facemask and −42.94% (from −44.24% to −41.60%) for distancing. When two or more interventions were implemented simultaneously, a greater decrease in the reproduction number of COVID-19 was observed [8]. Severe acute respiratory syndrome coronavirus 2 (SARS-CoV-2) was identified as the definitive infectious agent; however, social and environmental factors, such as air pollution, may also play a contributory role in the transmission of the virus in human population [9].

Fine particulate matter (particles with aerodynamic diameter equal to or less than 2.5 µm in diameter, PM_2.5_) may affect disease via a variety of mechanisms such as altering immune response, increasing oxidative stress, causing inflammatory injury, inducing mutagenicity and introducing respiratory tract damage [10,11,12]. Moreover, ambient air pollution was associated with various infectious outcomes, such as deaths due to lower respiratory infection [13], elevated fatality of severe acute respiratory syndrome (SARS) in China [14], increased risk of influenza [15], and upper respiratory infections incidence and hospital admission for respiratory infections [16]. In addition, SARS-CoV-2 can remain viable in aerosols for hours [17] and air particles are suspected to be capable of carrying the virus and facilitating its spread [18]. 

Table 1 summarizes current literature on the association between air pollution and COVID-19 outcomes. Studies in Northern Italy and among cities in China reported positive correlations between short-term exposure to PM_2.5_ and COVID-19 outcomes [19,20,21,22,23]. A Korean study concluded temporal association between COVID-19 incidence and other air pollutants, but not with PM_2.5_ [24]. Exposure to long-term PM_2.5_ was associated with COVID-19 mortality after controlling for different confounders [25,26,27,28,29,30]. In the United States, Wu et al. showed that each 1-µg/m^3^ increase in long-term PM_2.5_ exposure (2000–2016 annual average) was associated with 11% increase in COVID-19 mortality [25], which was also affected by the presence of other hazardous air pollutants [26]. Hendryx et al. showed a positive association between long-term PM_2.5_ and COVID-19 prevalence and fatality as of 31 May 2020, by applying a linear regression model [30]. Timely evidence on the association between long-term exposure to air pollution, especially PM_2.5_, and COVID-19 incidence is accumulating in the United States and in Europe based on arbitrary cutoff points of the pandemic. With the progression of COVID-19, more extensive data would allow us to examine whether COVID-19 incidence was associated with long-term exposure to PM_2.5_ during each surge of the pandemic and whether it might be modified by the implementation of preventive interventions, such as facemask mandates or stay home policies.

Therefore, we hypothesize that long-term PM_2.5_ exposure might lead to chronic respiratory inflammation, which in turn enhances risk of SARS-CoV-2 infection. Intervention policies, such as stay home orders and facemask mandates, might interrupt SARS-CoV-2 transmission and reduce risk of COVID-19. The purpose of this study is to test hypotheses that long-term exposure to PM_2.5_ may be associated with increased risk of COVID-19 and that this relationship might potentially be modified by facemask mandates and stay home orders. In this nation-wide ecologic study, we tested our hypotheses by investigating the relationship between COVID-19 incidence and long-term exposure to PM_2.5_, as well as facemask mandates and stay home orders, using the U.S. county-level data. The evaluation of these associations was based on the progression of the pandemic by analyzing two surges in the U.S. together and separately.

## 2. Materials and Methods

Data sources are summarized in Table 2. Specifically, county-level COVID-19 incidence data was obtained from Johns Hopkins University, Center for Systems Science and Engineering Coronavirus Resource Center (CSSE). County-level confirmed numbers of cases of 3261 counties across the U.S. have been updated daily utilizing the data from the CDC and state departments of health since 21 January 2020 [3]. The U.S. experienced two surges of incidence increases. The fewest 7-day average daily confirmed cases (20,764 cases on 28 May and 34,596 cases on 12 September) corresponded to the lowest point after each surge and thus marked the end of that surge. In this study, we used the cumulative incidence cases of COVID-19 reported in each county up to 28 May 2020, and up to 12 September 2020.

County-level annual average of PM_2.5_ between 2000 and 2016 as well as county-level covariates were available on a GitHub repository. The data utilized in the study assessing COVID-19 mortality and long-term exposure to PM_2.5_ was published by Wu et al. on this repository and was publicly available [25]. Briefly, van Donkelaar et al. estimated the ground-level concentration of PM_2.5_ using a chemical transport model and satellite observation calibrated using ground-level observation across North America [34]. 

County-level socioeconomic and demographic variables in 2016 were available from the US Census/American Community Survey. The 2020 behavioral factors such as prevalence of adult tobacco smoking and adult obesity were publicly accessible on the County Health Rankings & Roadmaps program, a program of the University of Wisconsin Population Health Institute aiming to provide a reliable and sustainable source of local data [31]. State average was used to replace missing values at county-level prevalence for smoking and obesity. State-wide policy of facemask use and stay home orders were collected and maintained by Boston University School of Public Health [5]. The New York Times summarized reopening policy for each state [32]. Up to date data on total tests performed in each state are available on the COVID tracking project [33]. A total of 165 counties were excluded because of the lack of a valid Federal Information Processing Standard code (*n* = 10) or missing covariates (*n* = 155). After exclusion, a total of 3096 counties were eligible and included in the study. 

This study aimed to estimate how COVID-19 incidence was associated with county-level long-term exposure to ambient PM_2.5_ and with state policies of facemask mandates and stay home orders during the first surge (as of 28 May 2020), during the second surge (between 28 May 2020, and 12 September 2020) and cumulatively (as of 12 September 2020). Since the COVID-19 temporal relationship with long-term exposure to PM_2.5_ is different from that with preventive interventions, separate negative binomial models were applied. Models were adjusted for potential confounders (Equations (1) and (2)).

(1) $$\begin{array}{ll} \log\left[E\left({\mathrm{Incidence}}_{i}\right)\right] & \\ & =\beta_{0}+{\;\beta }_{1}{\mathrm{PM}}_{2.5}+{\;\beta }_{2}\;\mathrm{populatoin}\;\mathrm{density}\;+{\;\beta }_{3}\;\mathrm{percentage}\;\mathrm{of}\;\mathrm{poverty}\\ & +{\;\beta }_{4}\;\mathrm{median}\;\mathrm{house}\;\mathrm{value}+{\;\beta }_{5}\;\mathrm{meidan}\;\mathrm{household}\;\mathrm{income}\\ & +{\;\beta }_{6}\;\mathrm{percentage}\;\mathrm{of}\;\mathrm{owner}\;\mathrm{occupied}\;\mathrm{property}+{\;\beta }_{7}\;\mathrm{percentage}\;\mathrm{of}\;\mathrm{African}\;\mathrm{American}\\ & +\beta_{8}\;\mathrm{percentage}\;\mathrm{of}\;\mathrm{Hispanic}+{\;\beta }_{9}\;\mathrm{percentage}\;\mathrm{of}\;\mathrm{population}\;\mathrm{less}\;\mathrm{than}\;\mathrm{high}\;\mathrm{school}\;\mathrm{education}\\ & +{\;\beta }_{10}\;\mathrm{smoke}\;\mathrm{rate}+{\;\beta }_{11}\;\mathrm{obese}\;\mathrm{rate}+{\;\beta }_{12}\;\mathrm{percentage}\;\mathrm{of}\;\mathrm{male}\\ & +{\;\beta }_{13}\;\mathrm{percentage}\;\mathrm{of}\;\mathrm{people}\;\mathrm{with}\;\mathrm{age}\;\mathrm{of}\;65\;\mathrm{and}\;\mathrm{above}\\ & +{\;\beta }_{14}\mathrm{duration}\;\sin\mathrm{ce}\;\mathrm{first}\;\mathrm{case}\;\mathrm{reported}+\beta_{15}\mathrm{reopening}+\beta_{16}\mathrm{pausing}\;\mathrm{reopened}\\ & +\beta_{17}\;\mathrm{total}\;\mathrm{tests}\;\mathrm{performed}+\beta_{18}\;\mathrm{facemask}\;\mathrm{mandate}+{\;\beta }_{19}\;\mathrm{days}\;\mathrm{of}\;\mathrm{stay}\;\mathrm{home}\;\mathrm{order}\\ & +\mathrm{offset}\left[\log\left(\mathrm{population}\right)\right]\; \end{array}$$

(2) $$\begin{array}{ll} \log\left[E\left({\mathrm{Incidence}}_{i}\right)\right] & \\ & =\beta_{0}+\beta_{1}{\mathrm{PM}}_{2.5}+{\;\beta }_{2}\;\mathrm{populatoin}\;\mathrm{density}+{\;\beta }_{3}\;\mathrm{percentage}\;\mathrm{of}\;\mathrm{poverty}\\ & +{\;\beta }_{4}\;\mathrm{median}\;\mathrm{house}\;\mathrm{value}+{\;\beta }_{5}\;\mathrm{meidan}\;\mathrm{household}\;\mathrm{income}\\ & +{\;\beta }_{6}\;\mathrm{percentage}\;\mathrm{of}\;\mathrm{owner}\;\mathrm{occupied}\;\mathrm{property}+{\;\beta }_{7}\;\mathrm{percentage}\;\mathrm{of}\;\mathrm{African}\;\mathrm{American}\\ & +\beta_{8}\;\mathrm{percentage}\;\mathrm{of}\;\mathrm{Hispanic}+{\;\beta }_{9}\;\mathrm{percentage}\;\mathrm{of}\;\mathrm{population}\;\mathrm{less}\;\mathrm{than}\;\mathrm{high}\;\mathrm{school}\;\mathrm{education}\\ & +{\;\beta }_{10}\;\mathrm{smoke}\;\mathrm{rate}+{\;\beta }_{11}\;\mathrm{obese}\;\mathrm{rate}+{\;\beta }_{12}\;\mathrm{percentage}\;\mathrm{of}\;\mathrm{male}\\ & +{\;\beta }_{13}\;\mathrm{percentage}\;\mathrm{of}\;\mathrm{people}\;\mathrm{with}\;\mathrm{age}\;\mathrm{of}\;65\;\mathrm{and}\;\mathrm{above}\\ & +{\;\beta }_{14}\mathrm{duration}\;\sin\mathrm{ce}\;\mathrm{first}\;\mathrm{case}\;\mathrm{reported}+\beta_{15}\mathrm{reopening}+\beta_{16}\mathrm{pausing}\;\mathrm{reopened}\\ & +\beta_{17}\;\mathrm{total}\;\mathrm{tests}\;\mathrm{performed}+\beta_{18}\;\mathrm{facemask}\;\mathrm{mandate}+{\;\beta }_{19}\;\mathrm{stay}\;\mathrm{home}\;\mathrm{order}\\ & +\beta_{20}\mathrm{incidence}\;14\;\mathrm{days}\;\mathrm{prior}+\mathrm{offset}\left[\log\left(\mathrm{population}\right)\right]\; \end{array}$$

County-level annual average of ambient PM_2.5_ between 2000 and 2016 was used as a measure for long-term exposure to PM_2.5_. Equation (1) was used to investigate the association between exposure to long-term PM_2.5_ and COVID-19. We adjusted for duration since the first case reported, population density, poverty, education, proportions of African Americans and Hispanic Americans, owner occupied property, median house value, median household income, gender, population older than 65 years old, and prevalence of tobacco smoking and obesity at county level and state-level variables, including policies of facemask mandates and the duration of stay home orders, total test results reported and reopening status. To examine the association between intervention policies and COVID-19 incidence as in Equation (2), facemask mandates and stay home orders were measured as binary variables. Their values as of 28 May 2020, were used to examine the association during the first surge and the values as of 12 September 2020, were used for the second surge and for both surges cumulatively. This also applied to other variables that changed over time, including duration since the first case reported, duration of stay home orders, total tests reported and reopening status. To address the potential reserve causation that policies might be a result of elevated COVID-19 incidence, we additionally controlled for COVID-19 incidence 14-days prior (as of 14 May 2020, for the first surge and as of 28 August 2020, for the second surge and for the cumulative analysis). To account for the correlation within each state, we applied the robust error estimation. Stratified analyses by facemask mandates and stay home orders were performed by applying Equation (1) to evaluate their effect measure modification on the association between PM_2.5_ and COVID-19 incidence. Relative risk (RR) and 95% confidence interval (CI) were reported. Analyses were performed in SAS 9.4 (SAS Institute Inc., Cary, NC, USA).

## 3. Results

A total of 3096 counties across the United States are included in this study and their characteristics are presented in Table 3. As of 12 September 2020, the average COVID-19 incidence was 2.60%, with a median of 1.27%. Counties with COVID-19 incidence greater than the national median had higher average annual PM_2.5_ concentration, earlier occurrence of the first case, more tests performed, and were less likely to be reopened. Higher population density, higher proportion of African Americans and Hispanic, population in poverty, population with less than a high school education and less owner-occupied properties were also observed in counties with increased incidence of COVID-19.

Table 4 shows the association found between COVID-19 infection and ambient PM_2.5_ after adjusting for potential covariates. Overall, each 1-µg/m^3^ increase in annual average concentration of PM_2.5_ was associated with 7.60% increase in the cumulative COVID-19 risk, with 95% CI between 3.82% and 11.51%. This association was consistent over two surges of the pandemic, with an increase from 5.06% to 8.58% for each 1-µg/m^3^ increase in PM_2.5_.

In counties eligible for this study, 1853 were located in states that had ever issued a facemask or face covering mandate. The RR of COVID-19 incidence for a county located within a state requiring facemask was 0.8462 (95% CI: 0.7592, 0.9433) as of 12 September 2020, after controlling for incidence case number 14 days prior (28 August 2020) and other covariates (Table 4). A similar association was observed during the second surge (between 28 May and 12 September 2020). However, facemask mandates seemed to have little impact on the incidence during the first surge (as of 28 May 2020). 

State-wide stay home policy (ever) was issued in 2659 counties. After adjusting for incidence of 28 August and other covariates, we observed a 7%-decrease in COVID-19 incidence among the counties with effective stay home policy, with a 95% CI between 0.8087 and 1.0604 (Table 4). Stay home policy showed similar protective effect during the second surge (RR = 0.9237, 95% CI: 0.7898, 1.0802) and this effect was stronger during the first surge (RR = 0.7615, 95% CI: 0.5619, 1.0321).

Since facemask mandates and stay home policy might be potential effect modifiers on the association between PM_2.5_ and COVID-19, we performed stratified analyses by facemask policy (ever issued/never issued) and by stay home policy (ever issued/never issued). Results are shown in Table 5. Though the incidence associated with 1-µg/m^3^ increase in PM_2.5_ seemed to be similar overall and during the first surge, this association was enhanced among counties locating in state with a facemask policy (RR = 1.1155, 95% CI: 1.0635, 1.1700) compared to those not requiring a facemask (RR = 1.0432, 95% CI: 0.9918, 1.0972). Those counties locating in a state without an effective stay home order experienced higher COVID-19 risk associated with 1-µ/m^3^ increase in PM_2.5_ (RR = 1.4050, 95% CI: 1.2961, 1.5230 for the first surge; RR = 1.1597, 95% CI: 1.1108, 1.2108 for overall), whereas slight increase was still observed overall (RR = 1.0795, 95% CI: 1.0384, 1.1223) and during the first surge (RR = 1.0186, 95% CI: 0.9565, 1.0848) in counties with an effective stay home order.

## 4. Discussion

Our study utilizing data up to 12 September 2020, from 3096 counties across the United States suggested that each 1-µg/m^3^ increase in long-term PM_2.5_ was associated with a 7.60% increase in COVID-19 incidence. Our data also suggested that preventive interventions, including facemask mandates and stay home orders, reduced the risk of COVID-19 by 15% and 8%, respectively. However, implementation of facemask mandates or stay home orders did not modify the association between long-term exposure to PM_2.5_ and COVID-19 incidence. The potential mechanisms for the impact of PM_2.5_ include (1) long-term exposure to PM_2.5_ might lead to chronic inflammation in the respiratory pathway, which predisposes individuals to COVID-19; (2) chronic exposure to PM_2.5_ might impair cilia, which acts as the first line of defense; as a result, people with abnormal cilia might be more vulnerable to any viral infection [34]; and (3) finally, PM_2.5_ exposure induces the over-expression of angiotensin-converting enzyme 2 (ACE2), which is the receptor SARS-CoV-2 binds to; this might also lead to increasing susceptibility to be infected [35].

These findings built on earlier findings by showing that long-term exposure to PM_2.5_ is a risk factor and by showing that the levels of exposure to PM_2.5_ in the U.S. are sufficiently high to increase the risk of COVID-19. Our results were consistent with the association between long-term exposure to PM_2.5_ and COVID-19 mortality. Wu et al. reported that each 1-µg/m^3^ increase in long-term PM_2.5_ was associated with 11% increase in COVID-19 mortality using the same exposure window and geographic location [25]. Our finding that long-term exposure to PM_2.5_ increased the risk of COVID-19 using the negative binomial models was consistent with the positive correlations reported by studies in Europe and in the U.S., employing different statistical models (Table 1) [27,28,29,30]. Thus, we provided an alternative perspective to examine such association when the linear assumption between COVID-19 incidence and PM_2.5_ concentration might not hold. 

Previously, a study in the U.S., using county-level data as of 31 May 2020, applied a linear regression model and suggested that an additional 23.5 COVID-19 cases were associated with each 1-µg/m^3^ increase in 2016 annual average PM_2.5_ concentration [30]. We confirmed the positive correlation and updated the COVID-19 incidence cases as of 12 September 2020. Other than using an arbitrary cutoff point, we examined the trend of the pandemic and selected the date corresponding to the end of each surge. Moreover, we applied a longer exposure window from 2000 to 2016 to better represent the long-term PM_2.5_ exposure than using a single-year average concentration. Potential bias due to disease progression was addressed by including additional confounders, such as days since the first case reported. In addition, we also consider the potential effect modification by facemask mandates or stay home orders. 

This is the first study to examine how the association between long-term PM_2.5_ exposure and COVID-19 incidence may be affected by state prevention policies, including facemask mandates and stay home policy. Importantly this study suggests a mitigation effect of stay home and face mask policies. Facemask mandates showed stronger protective effect toward later course of the pandemic (Table 4). This might be because the consciousness of wearing face coverings in public and the supply of face coverings increased as the pandemic progressed. Wearing facemasks is an effective way of preventing viral transmission via coughing droplets [7] and reduces infection of COVID-19 among health care workers [6]. Moreover, stay home order seemed to be more effective at the beginning of the pandemic (Table 4) and, as the virus spread slowed down, states tended to terminate such orders. This diluted the associations we observed for the later stage of the pandemic. Stay home orders, also known as lockdown, was associated with reduced air pollution in many countries [18,36,37,38,39,40,41], including the U.S. [38]. During lockdown, reduced overall mortality was observed in China [39]; less excess life cancer risk was estimated in India [40]; and saving due to reduced morbidity might meanwhile relieve economic loss [42]. Therefore, stay home orders might help to alleviate the burden of COVID-19 incidence via the reduction of virus transmission among individuals as well as reduced exposure to air pollution, which is a risk factor for COVID-19. 

The study was subject to several limitations. First, due to the nature of the ecologic study design, the results might be vulnerable to ecologic fallacy. In addition, we might still have residual confounding even after controlling for county-level and state-level covariates. Moreover, our exposure data for PM_2.5_ ends in 2016, which was 4 years before the pandemic. However, the previous exposure may still serve as an indicator for more recent ambient exposure and our result was consistent with the positive findings between short-term PM_2.5_ exposure and COVID-19 incidence previously reported [19,20,21,22]. Implementation of and compliance with facemask mandates and stay home policies might cause misclassification. However, this would dilute the effect and lead to more conservative estimates on the preventive effects. Though the incidence 14 days prior was controlled when assessing the association of policies, reverse causation might still be an issue, moving estimates towards the null. 

Further research should examine whether some of the elevated risk experienced by communities of color and low-income communities in the U.S. is due to higher exposure to air pollution. Potential policy implications of these findings include (1) the importance of further lowering the long-term exposure to PM_2.5_ in the U.S. and (2) the heightened importance of stay home and face mask policies among populations with air pollution exposure.

## 5. Conclusions

Our study added evidence that long-term PM_2.5_ exposure increased the risk of COVID-19 during each surge and cumulatively as of 12 September 2020, in the United States. Although both state-level implementation of facemasks mandates and stay home orders were effective in preventing the spread of COVID-19, no clear effect modification was observed with long-term exposure to PM_2.5_ on the risk of COVID-19.

## Figures and Tables

**Table 1:** Literature Review on Air Pollution and COVID-19.

Study Area	Study Period	Statistical Model	Findings
Northern Italy [19]	24 February 2020–13 March 2020	Recursive binary partitioning tree approach	Daily PM_10_ exceeding 50 µg/m^3^ with a 15-day lag was a significant predictor for COVID-19 incidence
Chinese cities (Wuhan, Xiaogan and Huanggang) [20]	25 January 2020–29 February 2020	Poison regression adjusting for other air pollutants and meteorological variables in each city	Daily PM_2.5_ was positively associated with COVID-19 incidence with RR from 1.036 to 1.144. The association with PM_10_ was negative with RR between 0.915 and 0.964. Results for other pollutants (SO_2_, CO, NO_2_, and 8-hour O_3_) were not consistent among the study sites.
Chinese cities (Wuhan and Xiaogan) [21]	26 January 2020–29 February 2020	Univariate linear regression	PM_2.5_ and NO_2_ were positively associated with COVID-19 incidence 4 days later in both cities, while PM_10_ and CO were inconsistent between cities.
120 Chinese cities [22]	23 January 2020–29 February 2020	Generalized additive model adjusting for meteorological variables with city fixed effects	PM_2.5_, PM_10_, NO_2_ and O_3_ with a 2-week lag were positively associated with COVID-19 incidence, while SO_2_ was negatively associated. A 10µg/m^3^ increase in PM_2.5_ with a 2-week lag was associated with a 2.24% increase in COVID-19 incidence.
49 Chinese cities [23]	As of 22 March 2020	Multivariate linear regression model adjusting for GDP per capita and hospital beds per capita	Both short-term (01/15/2020 – 02/29/2020) and long-term (2015–2019) exposure to elevated PM_2.5_ and PM_10_ were associated with increased COVID-19 fatality. A 0.24% and a 0.61% increase in COVID-19 fatality were associated with 10-µg/m^3^ increase in short-term and long-term PM_2.5_, respectively.
7 metropolitan cities and 9 provinces in Korea [24]	3 February 2020–5 May 2020	Generalized additive model adjusting for meteorological variables, location and date	Significantly temporal associations were observed between COVID-19 incidence and daily NO_2_, CO and SO_2_, but not with PM_2.5_, PM_10_ or O_3_.
3089 counties in the United States [25]	As of 18 June 2020	Negative binomial fixed model adjusting for 20 covariates	Each 1-µg/m^3^ increase in long-term PM_2.5_ exposure (2000–2016 annual average) was associated with 11% increase in COVID-19 mortality.
3223 counties in the United States [26]	As of 11 July 2020	Negative binomial fixed model adjusting for other pollutants as well as county characteristics	HAPs was associated with increase COVID-19 mortality. Each 1-µg/m^3^ increase in long-term PM_2.5_ exposure (2000–2014 annual average) was associated with 7% increase in COVID-19 mortality
355 municipalities in the Netherlands [27]	As of 5 June 2020	Linear regression controlling for covariates	Long-term exposure to PM_2.5_ and NO_2_ were positively associated with COVID-19 outcomes, including incidence and mortality, but not with SO_2_. Each 1-µg/m^3^ increase in long-term PM_2.5_ exposure (2015–2019) was associated with 9.4 more COVID-19 cases, 3.0 more hospital admissions, and 2.3 more deaths.
71 Italian provinces [28]	As of 27 April 2020	Spatial correlation	Positive correlations were observed between COVID-19 incidence and long-term exposure (2016–2019) to NO_2_, PM_2.5_, PM_10_ and O_3_.
20 Italian regions and up to 110 provinces [29]	As of 31 March 2020	Multiple linear regression	Both long-term exposure (2017 annual mean) to PM_2.5_ and PM_10_ were associated with COVID-19 incidence. Each 1-µg/m^3^ increase in PM_2.5_ was associated with 0.26 increase in base-10 transformed COVID-19 incidence.
3108 counties in the United States [30]	As of 31 May 2020	Linear regression with adjusting for county-level covariates	PM_2.5_ (2016 annual mean) and diesel PM were associated with both COVID-19 incidence and mortality. Additional 23.5 cases and 1.08 deaths were associated with each 1-µg/m^3^ increase in PM_2.5_.

**Table 2:** Summary of data sources.

Sources	Description
Johns Hopkins University Center for Systems Science and Engineering Coronavirus Resource Center (CSSE) [3]	Cumulative county-level confirmed cases up to 12 September 2020
GitHub repository by Wu et al. [25]	Annual average PM_2.5_ concentration between 2000 and 2016
The US Census/American Community Survey	County-level socioeconomic and demographic variables in 2016
The County Health Rankings & Roadmaps program [31]	Country-level behavioral variables in 2020
Boston University of Public Health [5]	State-level policy of face masks mandates and stay home orders
The New York Times [32]	State-level reopening policies
The COVID tracking project [33]	State-level total tests performed

**Table 3:** Characteristics of Counties (*n* = 3096) by COVID-19 Risk.

County Characteristics	Total (*n* = 3096)	COVID risk ≤ 1.29% (*n* = 1548)	COVID risk > 1.29% (*n* = 1548)
	Mean (SD)	Mean (SD)	Mean (SD)
Risk of COVID-19 as of 9/12 (%)	1.65 (1.60)	0.69 (0.34)	2.62 (1.78)
Average ambient PM_2.5_ (µg/m^3^) ^1^	8.40 (2.52)	7.49 (2.49)	9.32 (2.20)
Days since first case reported	163 (28)	156 (35)	170 (17)
Total test results reported by state (1000 tests)	2333 (2394)	2114 (2415)	2553 (2353)
Duration of stay at home issued by state	48 (40)	54 (44)	41 (35)
State stay-home order ^2^, *n* (%)			
Ever issued	2659 (85.89)	1312 (84.75)	1347 (87.02)
Never issued	437 (14.11)	236 (15.25)	201 (12.98)
State facemask policy ^2^, *n* (%)			
Ever issued	1853 (59.85)	964 (62.27)	889 (57.43)
Never issued	1243 (40.15)	584 (37.73)	659 (42.57)
State reopening status, *n* (%)			
Reopened	1225 (39.57)	815 (52.65)	410 (26.49)
Reopening	580 (18.73)	248 (16.02)	332 (21.45)
Pausing or reversing reopening plan	1291 (41.70)	485 (31.33)	806 (52.07)
Population density per square mile	427.39 (2184.38)	201.44 (720.43)	653.34 (2987.47)
African Americans population (%)	8.02 (14.07)	2.14 (5.07)	13.89 (17.35)
Hispanic Americans population (%)	7.54 (12.28)	5.14 (8.61)	9.94 (14.69)
Population living in poverty (%)	10.46 (5.90)	9.39 (5.36)	11.54 (6.20)
Population over 65 years old (%)	18.43 (4.50)	19.85 (4.28)	17.01 (4.27)
Male (%)	50.07 (2.20)	50.25 (1.93)	49.90 (2.43)
Population with less than high school education (%)	21.28 (10.68)	18.23 (9.53)	24.32 (10.90)
Owner occupied properties (%)	74.92 (8.41)	77.05 (6.94)	72.80 (9.18)
Median house value ($1000)	136.31 (91.08)	137.13 (88.39)	135.49 (93.71)
Median household income ($1000)	49.30 (13.41)	50.04 (11.87)	48.57 (14.75)
Ever smokers (%)	17.43 (3.54)	16.95 (3.44)	17.92 (3.57)
Obesity (%)	32.86 (5.41)	32.11 (5.09)	33.61 (5.62)

^1^ Annual average of PM_2.5_ between 2000 and 2016. ^2^ State stay-home order and facemask mandates ever issued before 12 September 2020.

**Table 4:** Adjusted RRs of COVID19 associated with 1-µg/m^3^ increase in PM_2.5_, facemask policy and stay home policy.

	RR (95% CI) (95% CI with Robust SE)		
	Surge 1 (as of 28 May 2020) ^3^	Surge 2 (between 28 May 2020 and 12 September 2020) ^4^	Cumulative (as of 12 September 2020) ^4^
PM_2.5_^1^	1.0506 (1.0269, 1.0747) (0.9857, 1.1197)	1.0852 (1.0696, 1.1011) (1.0361, 1.1366)	1.0756 (1.0612, 1.0901) (1.0376, 1.1149)
Facemask policy ^2^			
Never issued		Reference	
Ever issued	0.9889 (0.9180, 1.0652) (0.8667, 1.1283)	0.8360 (0.8030, 0.8704) (0.7298, 0.9577)	0.8466 (0.8166, 0.8776) (0.7598, 0.9432)
Stay home policy ^2^			
Never issued		Reference	
Ever issued	0.7615 (0.6928, 0.8370) (0.5619, 1.0321)	0.9168 (0.8664, 0.9701) (0.7833, 1.0730)	0.9193 (0.8734, 0.9677) (0.8021, 1.0537)

^1^ Model 1 adjusts for population density, poverty, education, proportions of African Americans, proportions of Hispanic Americans, owner occupied property, median house value, median household income, smoking prevalence, obesity prevalence, population over 65 years old, gender, days since first case reported, total test results, duration of safer at home policy, facemask policy, and reopening status. ^2^ Model 2 adjusts for all covariates in model 1 + incidence of COVID19 up to 14 days prior (14 May 2020 for surge 1 and 28 August 2020 for surge 2 and cumulative) and PM_2.5_. ^3^ State stay-home order and facemask mandates ever issued before 28 May 2020. ^4^ State stay-home order and facemask mandates ever issued before 12 September 2020.

**Table 5:** Adjusted RRs of COVID-19 associated with 1-µg/m^3^ increase in PM_2.5_ by facemask policy and by stay home policy.

	RR (95% CI) (95% CI with Robust SE)		
	Surge 1 (as of 28 May 2020) ^4^	Surge 2 (between 28 May 2020 and 12 September 2020) ^5^	Cumulative (as of 12 September 2020) ^5^
Face mask policy ^1^			
Never issued	1.0426 (1.0144, 1.0717) (0.9645, 1.1270)	1.0417 (1.0165, 1.0675) (0.9905, 1.0955)	1.0547 (1.0293, 1.0807) (1.0109, 1.1004)
Ever issued	1.0854 (1.0327, 1.1407) (0.9817, 1.2000)	1.1161 (1.0958, 1.1368) (1.0640, 1.1708)	1.0852 (1.0673, 1.1034) (1.0420, 1.1301)
Stay home policy			
Never issued ^2^	1.4050 (1.2885, 1.5319) (1.2961, 1.5230)	1.1056 (1.0406, 1.1746) (1.0478, 1.1665)	1.1543 (1.0870, 1.2257) (1.1016, 1.2095)
Ever issued ^3^	1.0186 (0.9947, 1.0431) (0.9565, 1.0848)	1.0970 (1.0803, 1.1140) (1.0441, 1.1526)	1.0798 (1.0648, 1.0949) (1.0386, 1.1226)

^1^ Model 1 adjusts for population density, poverty, education, proportions of African Americans, proportions of Hispanic Americans, owner occupied property, median house value, median household income, smoking prevalence, obesity prevalence, population over 65 years old, gender, days since first case reported, total test results, duration of safer at home policy, and reopening status. ^2^ Model 2 adjusts for population density, poverty, education, proportions of African Americans, proportions of Hispanic Americans, owner occupied property, median house value, median household income, smoking prevalence, obesity prevalence, population over 65 years old, gender, days since first case reported, total test results, facemask policy, and reopening status. ^3^ Model 3 adjusts for all covariates in model 2 + duration of safer at home. ^4^ State stay-home order and facemask mandates ever issued before 28 May 2020. ^5^ State stay-home order and facemask mandates ever issued before 12 September 2020.

## Data Availability

No new data were generated by the present study. All data analyzed are publicly accessible, as described in the method section, and their sources are listed in Table 2.
